# Novel Burst Suppression Segmentation in the Joint Time-Frequency Domain for EEG in Treatment of Status Epilepticus

**DOI:** 10.1155/2016/2684731

**Published:** 2016-11-02

**Authors:** Jaeyun Lee, Woo-Jin Song, Hyang Woon Lee, Hyun-Chool Shin

**Affiliations:** ^1^Department of Electrical Engineering, POSTECH, Pohang, Gyeongbuk, Republic of Korea; ^2^Department of Neurology, Ewha Womans University School of Medicine and Ewha Medical Research Institute, Seoul, Republic of Korea; ^3^Department of Electronic Engineering, Soongsil University, Seoul, Republic of Korea

## Abstract

We developed a method to distinguish bursts and suppressions for EEG burst suppression from the treatments of status epilepticus, employing the joint time-frequency domain. We obtained the feature used in the proposed method from the joint use of the time and frequency domains, and we estimated the decision as to whether the measured EEG was a burst segment or suppression segment by the maximum likelihood estimation. We evaluated the performance of the proposed method in terms of its accordance with the visual scores and estimation of the burst suppression ratio. The accuracy was higher than the sole use of the time or frequency domains, as well as conventional methods conducted in the time domain. In addition, probabilistic modeling provided a more simplified optimization than conventional methods. Burst suppression quantification necessitated precise burst suppression segmentation with an easy optimization; therefore, the excellent discrimination and the easy optimization of burst suppression by the proposed method appear to be beneficial.

## 1. Introduction

Electroencephalogram (EEG) burst suppression represents an inactivated EEG pattern, in which the aperiodic alternation of an isoelectric pattern (suppression) and a high voltage pattern (bursts) appears. The pattern found from an anesthetized cat's brain for the first time [1] would accompany the occurrence of a serious reduction in the brain's activity and metabolic rate [2], frequently seen from patients with postanoxic encephalopathy or status epilepticus under parenteral benzodiazepine treatments such as midazolam [3], those under general anesthesia [4], those with hypothermia [5], or those in a coma [6] or from neonates [7]. In the case of a burst suppression caused by anesthesia, the duration of the burst or suppression varies depending on the level of anesthetic concentration, with high levels identifying their relevance to a long duration of suppression [6, 8, 9]. In addition, a long duration of suppression has also identified its relevance with a worsening prognosis in certain cases (e.g., brain injuries caused by asphyxia), and, further, the progression of burst suppression has provided important prognostic information in previously conducted studies [10–12]. Accordingly, researchers have developed methods for quantification of burst suppression through calculations of the occupancy ratio of suppression in burst suppression (BSR) [13], analyses using the duration of suppression (interbursts interval, IBI) [14], and quantitation of a burst suppression probability [15].

The first step in quantifying the depth of burst suppression involves detecting the burst and suppression to distinguish them, thereby called burst suppression segmentation [16]. The current practice employed for this purpose involves a method based on visual detection, being a rough assessment and time-consuming task subjective to the viewers' interpretation. Thus, researchers initiated an algorithmic approach to burst suppression segmentation from the method used to manually set the threshold in an EEG pattern [13, 17, 18], evolving into the current methods in use. The methods of burst suppression segmentation developed thus far mainly involve detecting burst events by using certain features, such as Shannon entropy [19, 20], a nonlinear energy operator [21], line length [14], a voltage envelope [22], and variance using recursive-variance estimation [16]. These all represent features employed in the time domain, as well as occasionally the basic features of the frequency domain, like 3 or 10 Hz power or mean power spectral density (PSD) [19, 20, 23]. Previous studies also compared the performances by the basic features and segmentation methods [19, 20, 24].

In this study, we applied the joint use of features extracted from the time and frequency domains to burst suppression segmentation, to enhance the accuracy of segmentation conventionally conducted solely in the time domain. We thus set the EEG data and PSD thereof as the time-frequency vector to extract features to be used in the time-frequency domain. We then applied the Shannon entropy, the Tsallis entropy, and the regularity taken as features in broadly used conventional quantitative EEG (qEEG) analysis [25–28] to the time-frequency vector. After modeling of the distributions of burst and suppression of each feature into a Gaussian distribution, we employed the method of maximum likelihood estimation (MLE) to conduct the burst suppression segmentation. We assessed the accuracy of the proposed method by comparing the accordance to visually scored burst suppressions. In addition, we employed the BSR, as an indicator most widely used for quantification of burst suppression, and evaluated the closeness of estimated BSR to the true BSRs obtained from visual scores. In this study, the method presented revealed improved accuracy and precise BSR estimation. Besides, we solved the problems that reside in the optimization process of conventional methods through the application of Gaussian modeling and the MLE.

## 2. Methods

### 2.1. Data Acquisition

**Figure 1:**
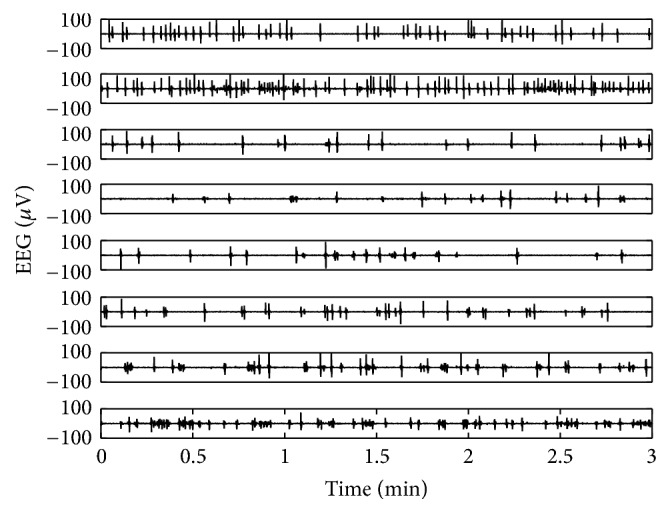
The different burst suppression characteristics in eight EEGs from patient number 1 (Table 1).

In this study, we used the 11 multichannel EEG data sets recorded from the Mokdong Hospital of Ewha Womans University. The patients were recruited based on the discharge database of the patients who had been hospitalized as status epilepticus and EEG monitoring was performed at the intensive care unit in Ewha Womans University Mokdong Hospital from July 2012 to June 2015. A total of 122 patients who met the criteria were enrolled so far, and their medical records for the age at onset, etiology, comorbidity, consciousness level before treatment, overall duration of status epilepticus, brain imaging, and visual interpretation of EEG were reviewed. Among them, four patients whose EEG clearly showed a burst suppression pattern were selected for the qEEG analysis in this study, using artifact-free EEG segments for at least 20 minutes chosen by an expert neurologist (H. W. Lee). Among those four patients with a burst suppression EEG pattern, three patients were male while one was female (male : female = 75% : 25%), and age ranged between 39 and 82 (mean: 61.5 ± 19.2) years (Table 1). Specifically, eight EEGs were recorded from patient number 1 (Table 1) on different days, while we obtained the rest of the three EEGs from three different patients.  Figure 1 shows the initial 3 minutes of the eight EEGs from patient number 1. We can observe the different burst suppression characteristics in the EEG evolution. The 11 EEGs were recorded from 21 electrode locations, based on the international 10–20 system with 200 Hz of sampling frequency. The 21 channels corresponding to each electrode were Fp1, Fp2, Fz, F3, F4, F7, F8, T3, T4, T5, T6, Cz, C3, C4, Pz, P3, P4, O1, O2, A1, and A2.

### 2.2. Time-Frequency Representation of Burst Suppression

**Figure 2:**
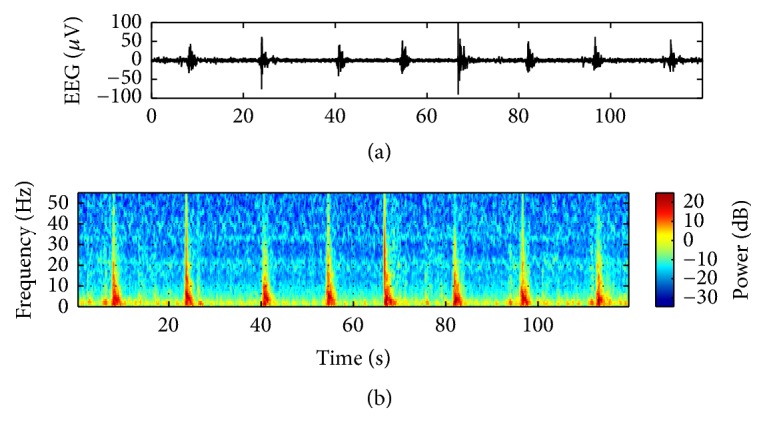
(a) Example of 120 s EEG burst suppression (median over all the channels) and (b) its PSD by spectrogram plot.

Let {*x*_*n*_(*i*) : *i* = 1,2,…, *L*} denote the raw sampled EEG signal of the *n*th channel. To remove artifacts in the EEG signals, we selected {*x*(*i*) : *i* = 1,2,…, *L*} with the median value over all channels. The median filtering over channels may lose time/frequency characteristics of the original EEG, but burst suppression patterns generally have synchrony over channels. Thus, the median value does not affect the burst suppression characteristics seriously as shown in Figure 2(a). Then, we obtained the power spectral density (PSD) of the EEG signal *x*(*i*). To accomplish this task, we defined the *m*th block of *x*(*i*) as follows:

(1) $$\begin{array}{c} x_{m}=\left\{\;x\left(\;i\;\right):i=1+m\Delta ,2+m\Delta ,\ldots ,N+m\Delta \;\right\}, \end{array}$$

where *N* indicates the block width and Δ means the sliding step. We calculated PSD **P**_*m*_ by the short-time Fourier transform (STFT) as follows:

(2) Xm=STFTxm,Pm=Xm2.

Figure 2 shows an example of EEG burst suppression patterns in both the time and the frequency domains. Note that we observed clear peaks corresponding to burst patterns in the frequency domain. This observation implied that we could detect the burst suppression patterns in the frequency domain, as well as in the time domain. Thus, we can expect that a joint analysis in both the time and the frequency domain may improve the accuracy of the burst suppression segmentation.

However, researchers execute most conventional segmentation or burst detection instances in the time domain. To do burst suppression segmentation in the time-frequency domain, we newly defined a joint time-frequency vector **f**_*m*_ as follows:

(3) $$\begin{array}{c} f_{m}=\left[\begin{array}{c} x_{m}\\ P_{m} \end{array}\right]. \end{array}$$

Then, we extracted entropy [19, 20, 25–27] and regularity [28] features (widely used in the time domain detection of qEEG) from **f**_*m*_.

### 2.3. Entropy and Regularity Features in the Time-Frequency Domain

Entropy serves as a method to quantify the order/disorder in signals, typically used for measuring the variety existing in burst suppression patterns. Most entropy-based EEG analyses use Shannon entropy [19, 20] or Tsallis entropy [25–27]. To calculate Shannon and Tsallis entropy, we first estimated the probability mass function (PMF),  *p*_*t*_ and *p*_*f*_ of **x**_*m*_ and **P**_*m*_, respectively, in (3). To estimate PMF, we introduced disjoint amplitude intervals *I*_*t*_(*k*) and *I*_*f*_(*k*), such that **x**_*m*_ = ⋃_*k*=1_^*k*=*M*_*t*_^ *I*_*t*_(*k*) and **P**_*m*_ = ⋃_*k*=1_^*k*=*M*_*f*_^ *I*_*f*_(*k*). Then, the probability that the signal belonged to the *k*th interval is the ratio between the number of the samples in the intervals and the total number of samples; that is,

(4) $$\begin{array}{c} \left[\begin{array}{c} p_{t}\left(k\right)\\ p_{f}\left(k\right) \end{array}\right]=\frac{1}{N}\left[\begin{array}{c} \text{The number of samples in }I_{t}\left(k\right)\\ \text{The number of samples in }I_{f}\left(k\right) \end{array}\right]. \end{array}$$

Then, we calculated Shannon entropy *S*(*m*) and Tsallis entropy *T*(*m*) as

(5) Sm=−∑l=1Mtptlln⁡ptl−∑l=1Mfpflln⁡pfl,Tm=1q−11−∑l=1Mtptlq1−∑l=1Mfpflq,

where *q* is a positive real value.

In addition, regularity could measure how smoothly or consistently the signals change. For the descending-ordered data *d*_*t*_(*i*) and *d*_*f*_(*i*) of **x**_*m*_^2^ and **P**_*m*_, respectively, we obtained the regularity of **f**_*m*_ in (3) as

(6) $$\begin{array}{c} R\left(\;m\;\right)=\left[\begin{array}{c} \sqrt{\frac{\sum_{i=1}^{N}i^{2}d_{t}\left(i\right)}{\left(N^{2}/3\right)\sum_{i=1}^{N}d_{t}\left(i\right)}}\\ \sqrt{\frac{\sum_{i^{\prime }=1}^{N}i^{2}d_{f}\left(i\right)}{\left(N^{2}/3\right)\sum_{i=1}^{N}d_{f}\left(i\right)}} \end{array}\right]. \end{array}$$

**Figure 3:**
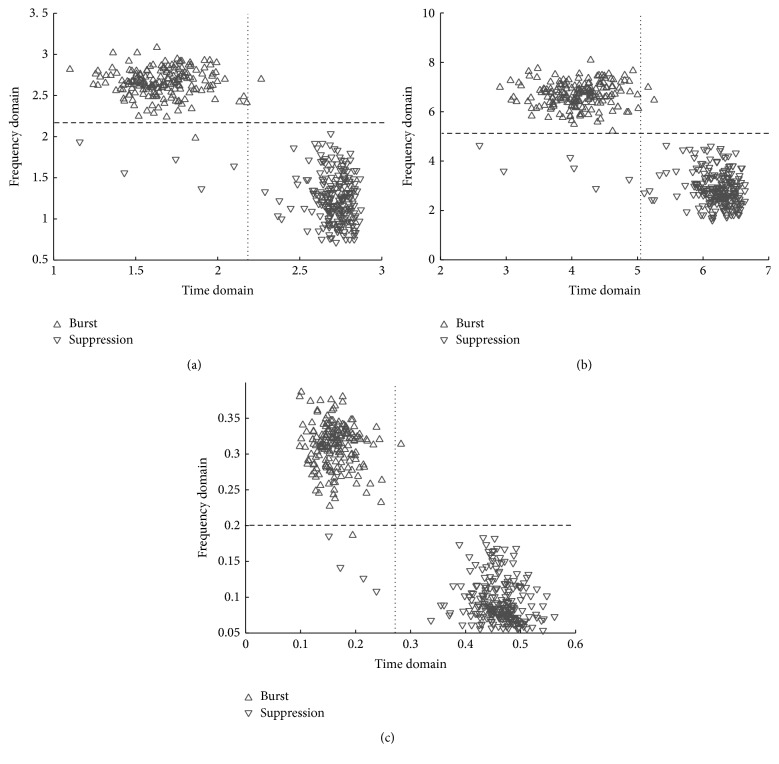
The example features of burst suppression in joint time-frequency domain. The features used are (a) Shannon entropy, (b) Tsallis entropy, and (c) regularity. Dotted line: a decision boundary of the sole use of time domain. Dashed line: a decision boundary of the sole use of frequency domain. The examined burst and suppression segments are identified by the experts.

The feature distributions of *S*(*m*),  *T*(*m*), and *R*(*m*) for the burst suppression pattern in the time-frequency domain are represented in Figures 3(a)–3(c), respectively, in which the patterns of burst and suppression corresponding to all three features are distinguished clearly. However, since some bursts and suppressions deviated from each pattern, the performance of the segmentation appeared to potentially be degraded thereby. By the single-domain approach, that is, the sole use of the time or frequency domain, the decision boundary would be a straight line. The vertical lines represented in Figures 3(a)–3(c) correspond to the decision boundary in the time domain, and, in this case, the errors of either a falsely alarmed burst or a missing burst could occur. The horizontal lines represented in Figures 3(a)–3(c) imply the decision boundary in the frequency domain and, in this case, could also be accompanied by misses and false alarms. Therefore, we took into account the use of a nonlinear decision boundary in the joint time-frequency domain, to improve the accuracy of the burst suppression segmentation. There exist many nonlinear classifiers, for example, artificial neural networks or support vector machines [19, 20]. Among these nonlinear classifiers, we employed the MLE, being the probabilistically optimal classifier.

### 2.4. Burst Suppression Segmentation

To conduct the MLE, we needed the corresponding probability distribution model. Thus, for this purpose, we modeled the distributions of features corresponding to the respective burst and suppression into the Gaussian distribution. Let the mean and covariance of a certain feature (i.e., one of the Shannon entropy, Tsallis entropy, and regularity) corresponding to burst patterns be ***μ***_*B*_ and **C**_*B*_, respectively. Similarly, let ***μ***_*S*_ and **C**_*S*_ be the mean and covariance of the features corresponding to suppression patterns. Then, we could express the Gaussian distribution model for bursts and suppressions as

(7) pBz=2π−1CB−1/2exp−12z−μBtCB−1z−μB,

(8) pSz=2π−1CS−1/2exp−12z−μStCS−1z−μS,

respectively, where |·| is the determinant of a matrix and (·)^*t*^ is the transpose of a vector.

Equations (7) and (8) denote the likelihood of the burst and suppression of feature **z**, and, in this study, we supposed the decision to maximize the likelihood. Let *θ* ∈ {*B*, *S*}; then, we expressed the decision rule as

(9) $$\begin{array}{c} \hat{\theta }=\underset{\theta }{arg\;max}\left\{\;p_{\theta }\left(\;z\;\right):\theta \in \left\{\;B,S\;\right\}\;\right\}. \end{array}$$

**Figure 4:**
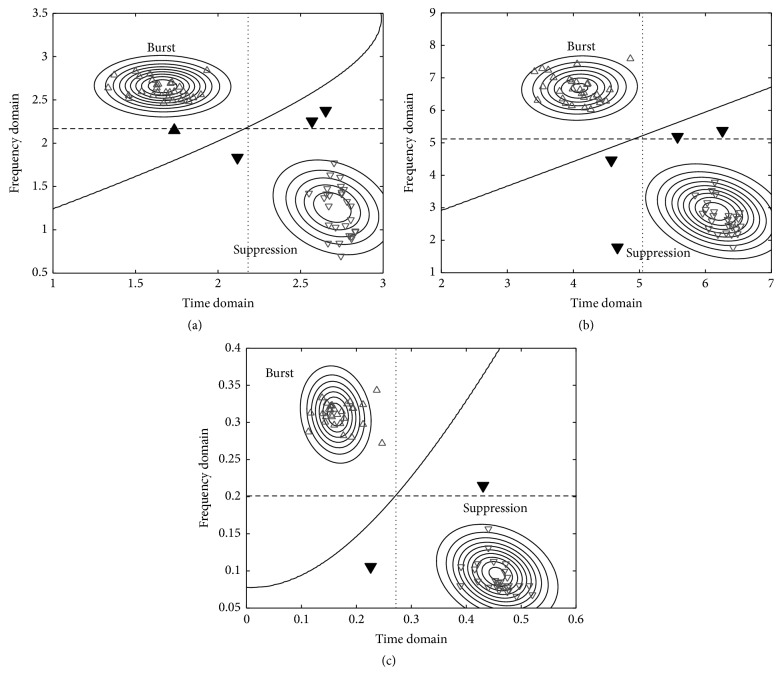
The example Gaussian models in the joint time-frequency domain. The features used are (a) Shannon entropy, (b) Tsallis entropy, and (c) regularity. The feature segments (triangles) are different segments from Figure 3. Solid line: the optimal decision boundary by MLE. Dotted line: the optimal decision boundary in the time domain solely. Dashed line: the optimal decision boundary in the frequency domain solely. Upward-pointing and downward-pointing empty triangles: detected true bursts and suppressions by all the boundaries. Upward-pointing filled triangle: a burst missed in time or frequency domain solely. Downward-pointing filled triangles: falsely alarmed suppressions in time or frequency domain solely.

Figure 4(a) represents Gaussian distributions of the Shannon entropy generated by (7) and (8) as the two concentric circles labeled as burst and suppression, respectively. For the Gaussian modeling, we used the data in Figure 3. Then, we represented the Shannon entropies of the burst and suppression obtained from the burst suppression in Figure 4(a) as upward-pointing triangles and downward-pointing triangles, respectively, all identified and distinguished clearly by the solid nonlinear line, the decision boundary determined by (9). In this case, we generated the false alarms and misses marked with filled triangle by the vertical and horizontal lines fixed at the optimal threshold. Specifically, the two filled downward-pointing triangles on the right side of Figure 4(a) represent errors generated by the misclassification of a suppression into a burst with the sole application of the frequency domain, while the filled downward-pointing triangle in the center represents an error of the misclassification of a suppression into a burst with the sole use of the time domain. The filled upward-pointing triangle on the left side of Figure 4(a) also represents an error owing to the misclassification of a burst into a suppression with the sole application of the frequency domain. Figures 4(b) and 4(c) represent the plots of cases using the Tsallis entropy and regularity, respectively. For these cases, (9) also rendered a clearly distinguished classification; however, we generated the miss or false alarm in cases of the sole use of either the time or the frequency domains.

### 2.5. Performance Evaluation

We derived the results of the burst suppression segmentation from the 11 consecutive EEGs recorded from 4 patients suffering from status epilepticus. We set the values of block width *N* and sliding step Δ as 140 and 40, respectively, implying the number of samples corresponding to 0.7 s and 0.2 s, respectively. The value *N* is chosen to include at most one burst segment, and the value Δ is chosen to be smaller than *N*/2. The frequency resolution involved in STFT calculation is chosen to be *N*, and this resolution was enough to identify the PSD distribution over the frequency axis. We set the values of *M*_*t*_ and *M*_*f*_ (the parameters used for the calculation of entropies) as 20 and 40, respectively, with *q* = 0.5 for Tsallis entropy. The parameters related to the features (i.e., *M*_*t*_,  *M*_*f*_, and *q*) were chosen to have enough divisibility of burst clusters and suppression clusters. From the EEGs of the total of at least 20 min each (mean 21.55 ± 0.61 min), we used 10 min duration to model the Gaussian distributions (7) and (8). Depending on the data, the number of bursts in 10 min duration varies. For sparse and dense bursting, about 80 and 220 burstings were observed in 10 min, respectively. Then, we used the latter half after the initial duration of ~10 min for the identification of the performance of the burst suppression segmentation.

To evaluate the overall accuracy of the burst suppression segmentation for the 11 EEG data sets, we employed the sensitivity, specificity, and accuracy based on the accordance to the visual scores [14, 21]. Sensitivity means the ratio of samples detected as true bursts among burst samples by the algorithm, and the specificity denotes the ratio of samples detected as true suppressions among suppression samples by the algorithm. The accuracy means the ratio of properly detected samples among whole samples, determined by taking the sensitivity and specificity into account. Therefore, the values for sensitivity, specificity, and accuracy increased along with the improved accuracy of the burst suppression segmentation.

As a result of the burst suppression segmentation, we could obtain the binarized burst suppression (BBS) pattern comprising sample units denoted as either 1 (burst sample) or 0 (suppression sample); and by using the BBS pattern, we could calculate the BSR, known most widely as the measure of burst suppression [15, 16, 19]. BSR is calculated as a ratio of the number of zeros in BBS in a certain interval to the number of samples in the interval, that is, a suppression ratio. Known also to be correlated with cerebral metabolism [9], the BSR can be used for various patient monitoring applications, including treatment of status epilepticus [29] and monitoring the depth of anesthesia [30]. We computed BSR at every 15-second interval with one sample sliding window. Then, we exploited the difference with visually scored BSR (true BSR) to identify the performance of burst suppression segmentation.

To evaluate the similarity between the true BSR and estimated BSR and to statistically analyze the results for all the EEG sets and all the features, we calculated the root-mean-square error (RMSE) between the true BSR and estimated BSR, as in the following:

(10) $$\begin{array}{c} RMSE=\sqrt{\frac{1}{L^{\prime }}\overset{L^{\prime }}{\underset{i=1}{\sum }}{\left(\text{true BSR}\left(i\right)-\text{estimated BSR}\left(i\right)\right)}^{2}}, \end{array}$$

where *L*′ is the number of BSR samples, being ~10 min duration for performance evaluation. A low RMSE for a feature means a good BSR estimation of the feature; therefore, statistically analyzed RMSE values can provide the usefulness of the segmentation methods.

By one visual score, the results can be meaningless. This is because there is no gold standard for burst suppression segmentation, and the results from a certain visual score are subjective. Therefore, to evaluate the performance of the proposed method, independent visual scores from more than one expert are needed. The visual scores in the results were from two clinical experts, and we exhibited agreement (i.e., sensitivity, specificity, and accuracy) and BSR estimation results for the two visual scores, rater #1 and rater #2 in Tables 2 and 4. In addition, we evaluated and exhibited interrater agreements in Table 3.

## 3. Results

### 3.1. Comparison with Time Domain Detection

**Figure 5:**
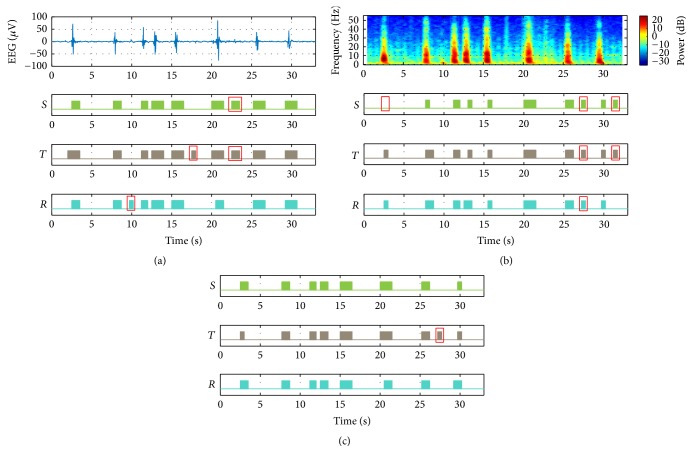
Example of segmentation results by various features: (a) a 33 s burst suppression (median value over all the channels) and detected bursts in the time domain using Shannon entropy (*S*), Tsallis entropy (*T*), and regularity (*R*); (b) PSD of (a) using a spectrogram plot (top) and detected bursts in the frequency domain, using Shannon entropy (*S*), Tsallis entropy (*T*), and regularity (*R*); (c) detected bursts in the joint time-frequency domain, using Shannon entropy (*S*), Tsallis entropy (*T*), and regularity (*R*). Blocks: detected bursts; boxes: false alarms and misses.

We represent the results of the burst suppression segmentation obtained from the sole use of the time domain for various features in Figure 5(a). We depicted the burst suppression pattern with a duration of 33 s on the top, and the plots placed thereunder and labeled as *S*,  *T*, and *R* represent intervals of burst detected as blocks, by using Shannon entropy, Tsallis entropy, and regularity, respectively. The intervals expressed as boxes in each plot represent all intervals identified as false alarms. Therein, we show one false alarm, two false alarms, and one false alarm corresponding to methods employing the Shannon entropy, Tsallis entropy, and regularity, respectively.  Figure 5(b) shows the results of the burst suppression segmentation obtained by the sole use of the frequency domain in the same burst suppression interval as that represented in Figure 5(a). The plot placed on the top represents the PSD portrayed as a spectrogram plot, and the plots placed thereunder represent the intervals of detected bursts, as in the case in Figure 5(a). In these plots, we expressed all the false alarms and misses as boxes and there evoked the one miss and two false alarms, the two false alarms, and the one false alarm, respectively, from the methods using the features of Shannon entropy, Tsallis entropy, and regularity. In Figure 5(c), we represent the results of the burst suppression segmentation obtained by the use of the proposed time-frequency domain in the same burst suppression interval. The methods using the Shannon entropy and regularity did not generate errors in the same interval; however, the method using the Tsallis entropy rendered one false alarm. Thus, as expected beforehand, the method using the proposed time-frequency domain resulted in reduced numbers of false alarms and misses generated.

**Figure 6:**
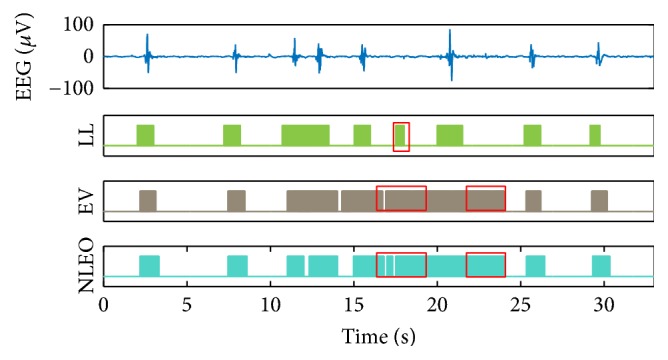
Example of 33 s EEG (median value over all the channels) and segmentation results of conventional methods; line-length- (LL-) based method, envelope- (EV-) based method, and nonlinear energy operator- (NLEO-) based method. Boxes: detected false alarms.

In Figure 6, we presented the interval of the bursts detected by conventional methods on the same burst suppression pattern presented in Figure 5. The conventional methods compared are those based on the line length (LL) [14], envelope (EV) [22], and nonlinear energy operator (NLEO) [21], all employed in detecting bursts. The plot placed on the top of Figure 6 represents the burst suppression, and the other three plots present bursts detected by conventional methods. In cases using the EV and NLEO methods, the false alarms evoked more than the case of the method using LL. In comparison to the proposed method in Figure 5, we identified that the burst suppression segmentation by the proposed method using the time-frequency domain could provide an improved accuracy.

Table 2 represents the mean and standard deviation (std) of the assessment indicators (i.e., sensitivity, specificity, and accuracy) for the whole 11 data sets, in correspondence with each feature. We represented the two features corresponding to the two respective highest assessment indicators in boldface. In general, the values for sensitivity tended to become higher with the method employing the time-frequency domain, in contrast to the values for specificity that appeared to remain relatively even. For rater #1, the highest accuracy appeared in the method employing the regularity with the time-frequency domain, and we obtained the second highest accuracy from the method based on LL. For rater #2, the two highest accuracies appeared in the proposed methods. For all three assessment indicators, the method using the time-frequency domain tended to show superior performance to that of the methods employing only the time domain.

Table 3 shows mean and std of sensitivity, specificity, and accuracy between two visual scores (rater #1 and #2). The sensitivity and specificity labeled by rater #1 versus rater #2 (true) were from the assumption that the visual score from rater #2 is truly segmented, and vice versa. The raters focused on picking out burst segments in burst suppression pattern, so the sensitivities between rater #1 and rater #2 can be significantly different. In Table 3, the sensitivity labeled as rater #1 versus rater #2 (true) is much smaller than the other sensitivity, and this means rater #2 was more sensitive to choose burst durations. Consequently, this difference between two visual scores implies that the performance evaluation by multiple raters is strongly recommended.

### 3.2. Burst Suppression Ratio (BSR)

**Figure 7:**
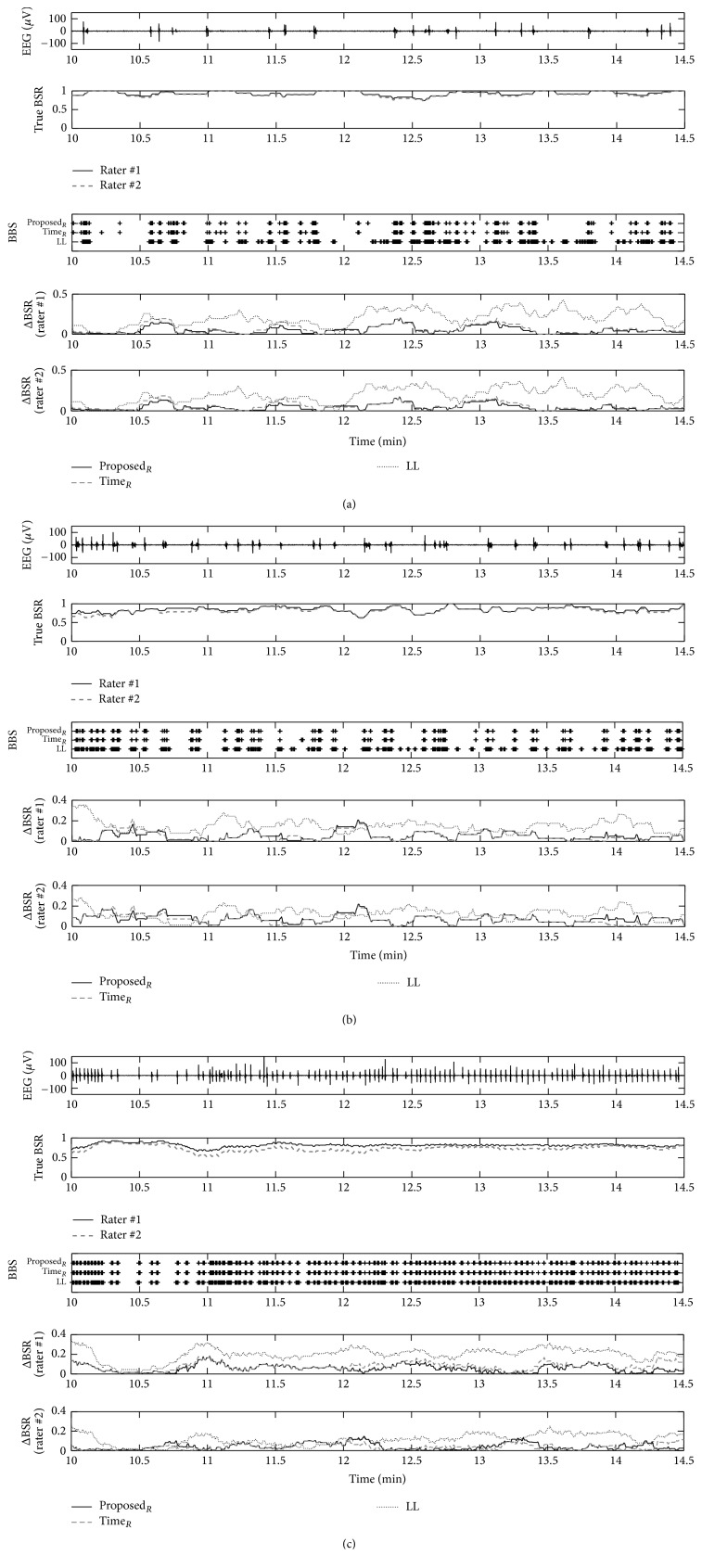
Three examples of burst suppression, segmentation results, and BSR estimates. Each subfigure shows burst suppression pattern, true BSR by visual segmentation of two raters, BBS which indicates detected bursts as bar plots, and ΔBSR progress for each true BSR. The features used are the proposed method using regularity (Proposed_*R*_), the time domain method using regularity (Time_*R*_), and LL-based method.

The first and the second plots in Figure 7(a) show the burst suppression pattern and the true BSRs from the visual scores of rater #1 and rater #2, respectively. The third plot therein represents the BBSs obtained, respectively, by the methods using the regularity in the time-frequency domain (Proposed_*R*_), employing the regularity in the time domain (Time_*R*_), and based on LL. The three features selected represent the ones evaluated as being excellent in terms of the accuracy. We represented the BBS plot with a marker on the points of 1 of the value of BBS generally corresponding to the points of the appearance of the burst.

To exhibit the exactitude of the estimated BSRs for each feature, we employed the absolute difference ΔBSR between the true BSR and estimated BSR (i.e., ΔBSR = |(true BSR) − (estimated BSR)|). The subfigures labeled as ΔBSR (rater #1) and ΔBSR (rater #2) of Figure 7(a) exhibited ΔBSRs regarding their true BSRs as of rater #1 and rater #2, respectively. With the distribution of low values for ΔBSR of Proposed_*R*_ in Figure 7(a), we can conclude that it represents the close estimation of the true BSRs. Figures 7(b)-7(c) represent the results of BSRs corresponding to different burst suppression patterns. The trend of the smaller value of true BSR from rater #2 than from rater #1 reflects the notion that rater #2 was more sensitive to choose burst durations. Through the three burst suppression patterns represented, the method employing the regularity with the proposed time-frequency domain shows the small ΔBSR.

Table 4 represents the mean RMSE and std over 11 EEG sets by each method of burst suppression segmentation. The lowest mean RMSE appeared from the regularity feature using the time-frequency analysis, and the next lowest one appeared from the regularity feature solely using the time domain, from both raters. Thus, the proposed burst suppression segmentation methods using the time-frequency analysis also rendered excellent results in terms of BSR estimation.

## 4. Conclusion

In this study, we exhibited the usefulness of exploiting time-frequency domain for burst suppression segmentation. Existing qEEG features are usually conducted in the time domain, but we newly redefined the features (i.e., Shannon entropy, Tsallis entropy, and regularity) in the time-frequency domain. These redefined two-dimensional features formed clusters of bursts and suppressions as in Figure 3. Because of the distribution of the clusters, the optimal classification must not be a sole use of the time or frequency domain. This means that the joint use of the time and frequency domain was expected to improve segmentation performance. To conduct the segmentation considering the distributions, we assumed that the clusters are modeled as Gaussians. Finally, using the Gaussian distributions, burst suppression segmentation is conducted by exploiting MLE, which is probabilistically optimal.

We compared the method developed in the time-frequency domain with the method employing the time domain only and with those of existing ones defined in the time domain, to derive and analyze the results of the burst suppression segmentation. Seeking precision, we used three assessment indicators comprising sensitivity, specificity, and accuracy for the comparison, and we improved the accuracy of the method proposed more than the sole use of the time domain. In addition, we also identified the accuracy of the proposed method similar to or better than that of existing methods defined in the time domain. To verify the usefulness of the proposed method, we employed BSR, which is broadly used as a measure of burst suppression and directly calculated by burst suppression segmentation, and evaluated the BSR for all compared methods. We evaluated the RMSEs of the true and estimated BSRs as indicators, and the regularity using the time-frequency domain revealed the best performance with the lowest mean RMSE among all methods compared with each other.

The method proposed in this study not only appears better than existing methods with respect to burst suppression segmentation, but also resolves the problems residing in the process of optimization of the conventional methods. The burst suppression pattern may vary according to the patients' condition or the environment of the measurement, so some conventional approaches with a fixed threshold can generate severe errors. However, in our study, we employed sufficient instances of burst and suppression segments in the burst suppression pattern for the Gaussian probabilistic modeling to solve such issues. In contrast to cases of existing algorithms using user-defined parameters optimized using ROC curves [14, 21, 22], for which the optimization process can be time-consuming as a result of redundant calculations of the sensitivity/specificity for slightly changing user parameters, the probabilistic modeling simplified and replaced this process. In conclusion, the improved accuracy and BSR estimation realized by the method proposed in this study demonstrated that burst suppression segmentation using the time-frequency domain appears to be more accurate and useful than that of conventional methods. However, in terms of data used, this study possesses a few shortages, which are the similar application (i.e., the treatment of status epilepticus) and the limited number of patients. The burst suppression pattern's occurrence has been reported in many applications, and compared methods actually have different applications (premature neonatal EEGs) from this study (treatments of status epilepticus). In fact, our data included multiple recordings in different days from one patient since the patient's medical condition had been changed every day during early hospital days, which could be a possible limitation of this study. By using more various applications of burst suppression and increased number of patients, we will be a little more confident about the improved performance of the proposed method. Besides, an application or introduction of new or different features to the method proposed in this study appears to be capable of bringing about even more promising performance.

## Figures and Tables

**Table 1:** Clinical manifestations of the patients.

Patient number	Age	Gender	Etiology	Diagnosis	EEG findings	Antiepileptic medications
1	39	M	Viral meningoencephalitis	Status epilepticus	Burst suppression	Midazolam IV^*∗*^
2	72	M	Cardiac arrest	Postanoxic encephalopathy	Burst suppression	None
3	82	F	Prolonged hypoxemia due to pneumonia	Postanoxic encephalopathy	Burst suppression	Midazolam IV^*∗*^
4	53	M	Viral encephalitis	Status epilepticus	Burst suppression	Midazolam IV^*∗*^

M: male; F: female; IV^*∗*^: intravenous continuous infusion.

**Table 2:** Mean (standard deviation) of sensitivity, specificity, and accuracy by different features and two raters, over the 11 EEGs.

	Features	Sensitivity (%)			Specificity (%)			Accuracy (%)		
		Rater #1	Rater #2	Avg.	Rater #1	Rater #2	Avg.	Rater #1	Rater #2	Avg.
Proposed time-frequency domain	Shannon entropy	68.55 (17.40)	78.22 (11.93)	73.39	88.66 (21.89)	82.78 (12.29)	**85.72**	84.51 (17.77)	82.44 (8.84)	**83.48**
	Tsallis entropy	69.81 (16.30)	74.03 (15.52)	71.92	88.31 (17.91)	81.20 (13.30)	84.76	83.29 (14.76)	80.73 (9.64)	82.01
	Regularity	74.42 (13.27)	91.81 (8.07)	**83.12**	88.60 (23.04)	82.50 (14.10)	85.55	84.82 (19.32)	85.20 (10.14)	**85.01**

Time domain	Shannon entropy	51.90 (15.10)	77.43 (11.29)	64.67	87.91 (7.03)	74.07 (14.94)	80.99	83.72 (9.01)	76.97 (9.14)	80.35
	Tsallis entropy	50.99 (14.35)	75.36 (13.05)	63.18	83.92 (6.02)	67.18 (13.28)	75.55	80.97 (8.31)	70.96 (8.09)	75.97
	Regularity	62.76 (11.46)	87.02 (7.42)	74.89	88.20 (7.97)	78.38 (16.71)	83.29	77.71 (8.08)	82.20 (10.41)	79.96

Conventional methods	Line length [14]	68.12 (31.30)	86.03 (5.66)	**77.08**	92.58 (12.89)	80.96 (12.72)	**86.77**	84.67 (13.42)	81.51 (9.45)	83.09
	Envelope [22]	57.13 (20.87)	75.62 (14.28)	66.38	82.83 (23.33)	77.22 (23.28)	80.03	77.32 (19.16)	76.48 (18.38)	76.90
	NLEO [21]	56.40 (21.90)	72.89 (14.75)	64.65	83.60 (22.17)	77.75 (24.70)	80.68	77.83 (18.70)	76.41 (18.87)	77.12

*Note*. Avg.: average between two raters; boldface: the two highest means in Avg. columns.

**Table 3:** Interrater sensitivity, specificity, and accuracy (mean ± standard deviation over the 11 EEGs).

	Sensitivity (%)	Specificity (%)	Accuracy (%)
Rater 1 versus Rater 2 (true)	81.26 ± 7.67	99.30 ± 0.80	95.34 ± 3.26
Rater 2 versus Rater 1 (true)	97.74 ± 1.20	94.56 ± 4.29	

**Table 4:** RMSE between true BSR and estimated BSR by burst suppression segmentation methods and two raters.

	Features	RMSE between true BSR and estimated BSR (mean ± standard deviation over the 11 EEGs)	
		Rater #1	Rater #2
Proposed time-frequency domain	Shannon entropy	0.111 ± 0.074	0.121 ± 0.070
	Tsallis entropy	0.121 ± 0.068	0.125 ± 0.068
	Regularity	**0.094** ± **0.051**	**0.104** ± **0.063**

Time domain	Shannon entropy	0.118 ± 0.067	0.134 ± 0.064
	Tsallis entropy	0.131 ± 0.058	0.142 ± 0.054
	Regularity	0.098 ± 0.049	0.113 ± 0.049

Conventional methods	Line length [14]	0.175 ± 0.053	0.152 ± 0.095
	Envelope [22]	0.210 ± 0.136	0.198 ± 0.148
	NLEO [21]	0.214 ± 0.130	0.196 ± 0.161

*Note*. Boldface: the lowest means in column.
